# Potential syntrophic relationship between coral-associated *Prosthecochloris* and its companion sulfate-reducing bacterium unveiled by genomic analysis

**DOI:** 10.1099/mgen.0.000574

**Published:** 2021-05-05

**Authors:** Yu-Hsiang Chen, Shan-Hua Yang, Kshitij Tandon, Chih-Ying Lu, Hsing-Ju Chen, Chao-Jen Shih, Sen-Lin Tang

**Affiliations:** ^1^​ Bioinformatics Program, Taiwan International Graduate Program, National Taiwan University, Taipei, Taiwan, ROC; ^2^​ Bioinformatics Program, Institute of Information Science,Taiwan International Graduate Program, Academia Sinica, Taipei, Taiwan, ROC; ^3^​ Biodiversity Research Center, Academia Sinica, Taipei, Taiwan, ROC; ^4^​ Institute of Fisheries Science, National Taiwan University, Taipei, Taiwan, ROC; ^5^​ Institute of Molecular and Cellular Biology, National Tsing Hua University, Hsinchu, Taiwan, ROC; ^6^​ Molecular and Biological Agricultural Sciences, Program Taiwan International Graduate Program, National Chung Hsing University and Academia Sinica, Taipei, Taiwan, ROC; ^7^​ Graduate Institute of Biotechnology, National Chung Hsing University, Taichung, Taiwan, ROC; ^8^​ Bioresource Collection and Research Center, Food Industry Research and Development Institute, Hsinchu, Taiwan, ROC

**Keywords:** *Prosthecochloris*, *Halodesulfovibrio*, *Isopora palifera*, Endolithic bacteria, Coral-associated bacteria, Coral-associated *Prosthecochloris*

## Abstract

Endolithic microbial symbionts in the coral skeleton may play a pivotal role in maintaining coral health. However, compared to aerobic micro-organisms, research on the roles of endolithic anaerobic micro-organisms and microbe–microbe interactions in the coral skeleton are still in their infancy. In our previous study, we showed that a group of coral-associated *Prosthecochloris* (CAP), a genus of anaerobic green sulphur bacteria, was dominant in the skeleton of the coral *Isopora palifera*. Though CAP is diverse, the 16S rRNA phylogeny presents it as a distinct clade separate from other free-living *Prosthecochloris *. In this study, we build on previous research and further characterize the genomic and metabolic traits of CAP by recovering two new high-quality CAP genomes – *Candidatus* Prosthecochloris isoporae and *Candidatus* Prosthecochloris sp. N1 – from the coral *I. palifera* endolithic cultures. Genomic analysis revealed that these two CAP genomes have high genomic similarities compared with other *Prosthecochloris* and harbour several CAP-unique genes. Interestingly, different CAP species harbour various pigment synthesis and sulphur metabolism genes, indicating that individual CAPs can adapt to a diversity of coral microenvironments. A novel high-quality genome of sulfate-reducing bacterium (SRB)– *Candidatus* Halodesulfovibrio lyudaonia – was also recovered from the same culture. The fact that CAP and various SRB co-exist in coral endolithic cultures and coral skeleton highlights the importance of SRB in the coral endolithic community. Based on functional genomic analysis of *Ca*. P. sp. N1, *Ca*. P. isoporae and *Ca*. H. lyudaonia, we also propose a syntrophic relationship between the SRB and CAP in the coral skeleton.

## Data Summary

All sequencing data and assembled genomes are available through National Center for Biotechnology Information (NCBI) repositories under BioProject ID: PRJNA595808. Sequence reads of metagenomes from endolithic culture can be found under SRA accession numbers SRR10714424, SRR10714423, SRR10714422, and SRR10714421, respectively.

**Impact Statement**

Little is known about the ecological roles of endolithic microbes in the coral skeleton; one potential role is as a nutrient source for their coral hosts. Here, we identified a close ecological relationship between CAP and SRB. Recovering novel high-quality CAP and SRB genomes from endolithic cultures in this study enabled us to understand the genomic and metabolic features of anaerobic endolithic bacteria in coral skeletons. These results demonstrate that CAP members with similar functions in carbon and nitrogen metabolisms harbour different light-harvesting components, suggesting that CAP in the skeleton adapts to niches with different light intensities. Our study highlights the potential ecological roles of CAP and SRB in coral skeletons and paves the way for future investigations into how coral endolithic communities will respond to environmental changes.

## Introduction

Microbial symbionts in reef-building corals, which support a variety of marine life, reside in the mucus, tissue and skeleton of diverse corals, influencing health of its host coral [1, 2]. Microbial symbionts comprise bacteria, archaea, algae, fungi and viruses, and their composition is influenced by their host corals’ genetic factors and dynamic environmental conditions [3]. They can help corals prevent or mitigate diseases and benefit corals by involving them in carbon, nitrogen and sulphur cycles [4]. For example, coral-dominant dinoflagellate *Symbiodiniaceae* can fix carbon dioxide and provide corals with organic compounds [5]. On the other hand, *Cyanobacteria* can fix nitrogen and provide the coral *Montastraea cavernosa* with a nitrogen source [6].

Compared to aerobic micro-organisms, the role of anaerobic micro-organisms in coral is not well understood. Previous studies found green sulphur bacteria (GSB) in a wide range of corals, including *Porites lutea*, *Platygyra carnosa*, *Montastraea faveolata* and *Montipora venosa* [7–10]. In addition, our previous study found that *Prosthecochloris *, a GSB genus, was dominant in skeletons of the coral *I. palifera*, forming a distinct green colour region beneath the coral tissue [11], although the algae *Osterobium* were previously thought to be the main microbial contributor to coral green layers [11–13]. Moreover, nutrients generated from micro-organisms in the coral skeleton were shown to be potential alternative sources of energy and nutrients [14, 15]. Therefore, the *Prosthecochloris* dominant in green layers may also be associated with stony coral health.

Most GSB are obligate anaerobic photoautotrophic bacteria that use the reverse tricarboxylic acid (rTCA) cycle to fix carbon dioxide [16]. During photosynthesis, the majority of them utilize reduced sulphur compounds as electron donors, while some – including *Chlorobium ferrooxidans* and *C. phaeoferrooxidans –* use ferrous iron [17–19]. Furthermore, some GSB are capable of obtaining reduced sulphur compounds through a syntrophic interaction with sulphur‐reducing bacteria (SRB), such as *Desulfuromonas acetoxidans* [20]. On the other hand, many GSB can fix nitrogen gas, which they use for growth [16]. GSB are found in various anoxic environments – including freshwater, hot springs, and seawater – and some of them are adapted to light-limited environments [16]. Among GSB, *Prosthecochloris* is mainly present in marine environments and has the ability to tolerate high salinity [16].

Though *Prosthecochloris* and most other GSB have been isolated as free-living bacteria [16], our previous study used amplicon and whole-metagenome analyses and found that *Prosthecochloris* is dominant in green layers of coral *I. palifera* skeletons, suggesting that the bacteria can interact with eukaryotic hosts and various bacteria [11, 13]. Through a phylogenetic analysis of the 16S rRNA gene, we found that, although *Prosthecochloris* from coral were diverse, they could be classified into a monophyletic clade separate from other free-living *Prosthecochloris *. Hence, we proposed a group of coral-associated *Prosthecochloris* (CAP) [11]. Furthermore, based on a gene-centric metagenome analysis, we proposed that CAP can fix nitrogen and nutrient cycling occurs in the coral skeleton.

The role of endolithic microbiomes in the coral reef system has been overlooked [21]. To provide detailed insights into the ecological roles of CAP and microbe–microbe interactions in the coral skeleton, high-quality genomes of endolithic microbes are needed. The genome for the CAP *Candidatus* Prosthecochloris A305, which we identified by metagenome-binning, is only 79 % complete. Other metagenomic bins identified were highly contaminated with other species. These results hindered our understanding of the metabolic features of CAP and illuminated syntrophic relationships between CAP and other micro-organisms in the coral skeleton.

Using an anaerobic culture approach, three endolithic cultures dominated by CAP were successfully obtained. The cultures, containing purer and more simplified communities and sufficient genomic DNA, enabled us to obtain the high-quality genomes of CAP and other companion bacteria using a whole-metagenome sequencing approach. In this study, we recovered two high-quality CAP genomes from the metagenomes of the coral endolithic cultures. These new genomes allowed us to compare functional genomic and phylogenetic features in CAP and to elucidate its diversity. In addition, based on our long-term observation for the skeleton of coral *I. palifera*, there are green and green-brown colours in the green layer beneath coral tissues, suggesting that there is habitat specificity of different GSB groups in coral skeleton. Besides GSB, we also identified a novel, predominant SRB genome from the same cultures. Based on functional genomic analysis in these genomes, we propose a syntrophic relationship between CAP and SRB in the coral skeleton.

## Methods

### Sample collection and anaerobic endolithic culturing

Three *I. palifera* colonies were collected from the ocean near Gongguan (22°40′ N 121°27′ E) in Lyudao, Taiwan (also known as Green Island) on 16 October 2017. These colonies were placed in an anaerobic jar with an anaerobic pack immediately after sampling. Green layers from each colony were collected as described in our previous studies [11, 13]. The anaerobic condition was maintained throughout the collection process. Bacteria in the green layers were enriched in the basal medium for *Prosthecochloris *, which consisted of 0.5 g l^−1^ KH_2_PO_4_, 5.3 g l^−1^ NaCl, 0.5 g l^−1^ MgSO_4_-7H_2_O, 0.7 g l^−1^ NH_4_Cl, 0.33 g l^−1^ KCl, 21 g l^−1^ Na_2_SO_4_, 4.0 g l^−1^ MgCl_2_-6H_2_O, 10 g l^−1^ NaHCO_3_, 0.07 g l^−1^ CaCl_2_-2H_2_O and 0.005 g l^−1^ Resazurin, and supplemented with glucose (0.05%) as an additional carbon source [11, 22]. The entire culturing process was performed under dim light (45.5 ± 31.5 lums/ft^2^) conditions.

### DNA extraction and whole-genome shotgun sequencing

Bacterial cells in the culture medium were centrifuged at 7 000 ***g*** for 10 min at 20 °C to obtain cell pellets. Total genomic DNA from the pellet was then extracted using the UltraClean Microbial DNA Isolation Kit (MioBio, Solana Beach, CA, USA) according to the manufacturer’s protocol and DNA concentration was determined by Nanodrop and Qubit. The DNA samples were sent to Yourgene Bioscience (Taipei, Taiwan) for library preparation and DNA sequencing by the Illumina MiSeq system (USA) with 2×300 cycles.

### Metagenome assembly and binning

Reads obtained from Illumina MiSeq were quality checked by FastQC [23]. Quality trimming and removal of Illumina adaptors were performed by Trimmomatic v0.39 with the following parameters: ILLUMINACLIP:TruSeq3-PE-2.fa:2 : 30 : 10 : 3: TRUE LEADING:10 TRAILING:10 SLIDINGWINDOW:5 : 15 MINLEN:50 CROP:300 [24]. Leading and trailing bases with Phred quality score<15 were trimmed using a 5-base wide sliding window. Only reads with >50 bases were retained. The processed reads from three cultures were *de novo* assembled individually using megahit with *k*-mer sizes of 21, 31, 41, 51, 61, 71, 81, 91 and 99 [25] without scaffolding. Automated binning was performed using MetaBAT v0.32.5 with default settings, which reconstructed genomes from assembled metagenomic contigs based on probabilistic distances of genome abundance and tetranucleotide frequency [26].

### Quality assessment, taxonomic inference, and relative proportion of MAGs

The quality of each metagenome-assembled genome (MAG) was accessed by CheckM v1.0.13, which uses lineage-specific marker genes to estimate completeness and contamination [27]. The taxonomy of each MAG was automatically assigned by GTDB-Tk v0.3.2 based on the placement of the genome in the reference tree, average nucleotide identity (ANI) values, and relative evolutionary divergence (RED) values [28]. To estimate the relative proportion of MAGs in each culture, reads were first mapped to assembled contigs using Bowtie2 v2.3.5 [29] with default settings. Results of the mapped reads were then used to obtain coverage for each contig and the relative proportion of each MAG with the ‘coverage’ and ‘profile’ command in CheckM, respectively.

### Genome annotation

The genome of CAP and *Candidatus* Halodesulfovibrio lyudaonia were annotated using Prokka v1.13.7 with the ‘usegenus’ and ‘rfam’ options [30]. The genomes were also annotated with KEGG functional orthologs (K numbers) by searching the putative protein sequences from Prokka against the KEGG database using BlastKoala [31]. The K number annotation results were then used to reconstruct the transporter systems and metabolic pathways using KEGG mapper [32]. Additionally, the transporter proteins were identified by searching for the putative protein sequences against TransportDB 2.0 (August 2019) using blastp [33].

### Recruitment of contigs with 16S rRNA gene sequences

The contigs with 16S rRNA gene sequences were originally not binned into the draft genome. To recruit the 16S rRNA gene, blastn was used to identify the contigs with *Prosthecochloris *-related 16S rRNA genes with an identity of >97 %. Only one *Prosthecochloris *-related 16S rRNA gene was identified in each culture, consistent with the finding that only one CAP genome was recovered. Based on these results, each contig containing *Prosthecochloris* 16S rRNA gene was moved into the CAP draft genomes.

### ANI calculation and phylogenetic analysis

The ANIs between genomes were determined using the ANI calculator [34] and the ANI matrices were visualized using the pheatmap function [35] in R (R core team, 2016). To analyse the 16S rRNA gene phylogeny of *Chlorobiaceae* and *Halodesulfovibrio *, the available *Chlorobiaceae* genomes and representative *Desulfovibrio* genomes were retrieved from the RefSeq database (August 2019) [36] and 16S rRNA gene sequences in the genomes were extracted by Barrnap v0.9 [37]. On the other hand, *Halodesulfovibrio* 16S rRNA gene sequences were downloaded from the NCBI 16S rRNA database and included in the analysis. A multiple sequence alignment of these 16S rRNA genes was performed using muscle [38], followed by a tree reconstruction by the maximum-likelihood method based on the Jukes-Cantor model and initial tree generation using the BioNJ method in mega7 [39, 40]. The confidence levels of the tree were determined using 1000 bootstraps [41].

For the FMO phylogeny, the FMO proteins were retrieved from the available *Chlorobiaceae* genomes in RefSeq database [36]. A tree was then inferred using the maximum-likelihood method based on the JTT matrix-based model [42] and initial tree generation using the BioNJ method in mega7 [39] with 1000 bootstraps.

A tree was built from single-copy marker genes using the ezTree pipeline [43]. Briefly, the putative genes in the genomes were identified by Prodigal [44], and the Pfam profiles of these genes were annotated using HMMER3 [45]. Gene annotations were compared to identify single-copy marker genes among the input genomes. The amino acid sequences of single-copy marker genes were then aligned by muscle [38]. The alignments were trimmed using Gblocks [46], and a tree based on the concatenated alignment was constructed by maximum-likelihood using FastTree with 1000 bootstraps [41, 47].

### Pan-genome analysis

Bacterial Pan Genome Analysis tool (BPGA) v1.3 [48] was used to perform a pan-genome analysis. The genes in the *Prosthecochloris* genomes were first clustered using USEARCH [49] with a 70 % identity cutoff. Gene clusters present in all the genome were defined as core genes, and those present in at least two – but not all – of the genomes were defined as accessory genes. The representative sequences of CAP-specific accessory genes were then searched against the NCBI RefSeq database [36] to identify the potential orthologous genes in bacteria, with 40 % identity and 50 % alignment length cutoffs. In addition, the d*N*/d*S* values of each CAP-unique accessory gene were determined using the HyPhy tool in mega7 [39].

## Results

### Diverse color bands in the green layers of coral skeletons

**Fig. 1.**
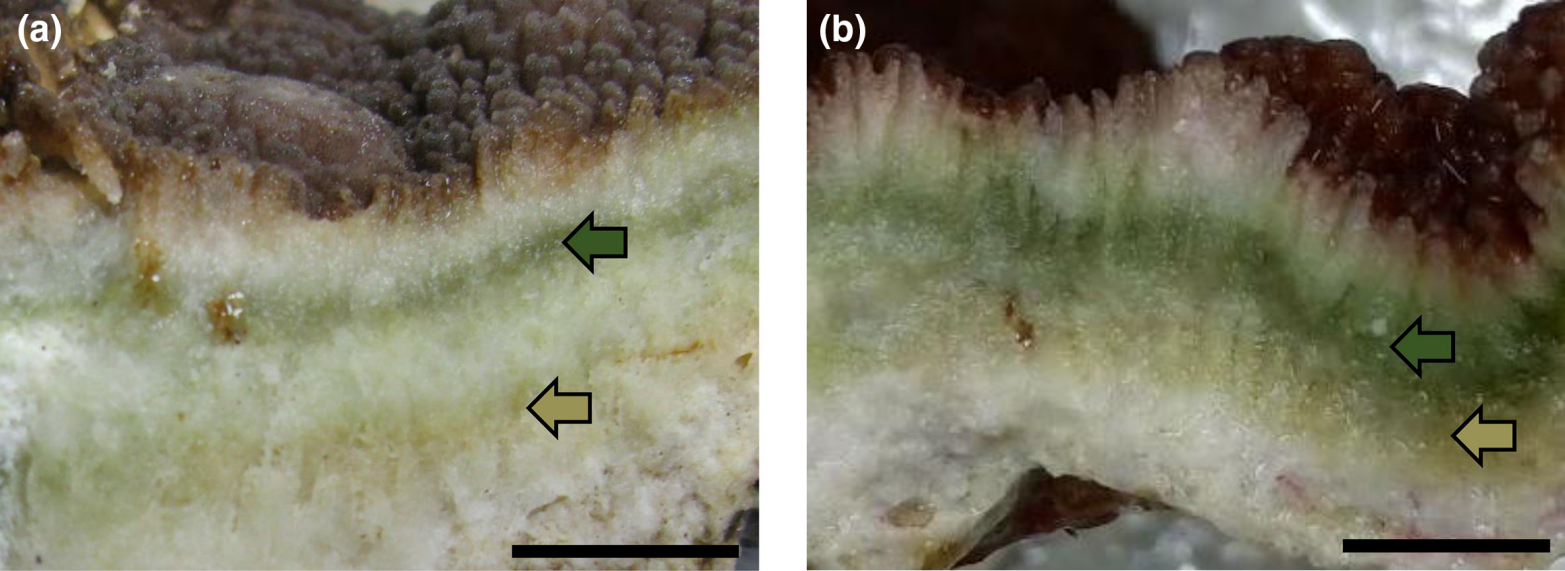
Comparison of skeleton of *I. palifera* collected from different years shows green and brown-green colours constantly stratified. Skeletons of *I. palifera* were collected and observed from Gongguan on 25 April 2014 (a) and 14 March 2020 (b). Scale bars represent 1 cm.

The investigation of coral skeleton from two *I. palifera* colonies was conducted in 2014 and 2020 from Gongguan (Lyudao, Taiwan). The cross‐sectional view of the samples revealed diverse colour in the green layers of *I. palifera* (Fig. 1). The region of skeletons close to the tissue were green, while the region close to white layers appeared green-brown. The spatially heterogeneous distribution of pigments indicated that microbial communities could be differentially distributed inside the green layers. The similar observations in different coral colonies, coral reefs and sampling time indicated that the phenomenon appears to be common. In order to identify and characterize the two CAP groups, the bacteria in the green layers were enriched using anaerobic culture techniques, and whole-genome shotgun sequencing was performed.

### High-quality bins recovered from coral endolithic cultures

Three colonies were collected to perform three coral endolithic cultures (N1, N2 and N3). Reads from N1, N2 and N3 cultures were individually *de novo* assembled and binned, yielding five, five and four bins, respectively (Table 1). Bins from cultures had similar taxonomic profiles, dominated by *Prosthecochloris *-related bins in N2 and N3 and *Ilyobacter *-related bins in N1 (Table S1). On the other hand, *Halodesulfovibrio *-related bins were the most abundance sulfate-reducing bacterial bins in the three coral endolithic cultures. Other genera represented in bins were *Marinifilum, Pseudovibrio* and *Desulfuromonas *, which were present in two of the three cultures. Among the total 14 bins identified, nine were high-quality (>90 % complete and <5 % contamination). The *Prosthecochloris *-related bins, including Bin ID N1-5, N2-4, and N3-4, had particularly high quality (>98.8 % complete) and low contamination (<1.5 %); *Halodesulfovibrio *-related bins in N3 was also high-quality (99.41 %) with very low contamination (0.26 %) (Table 1). Both *Prosthecochloris-* and *Halodesulfovibrio-*related bins lacked strain heterogeneity, suggesting that the bins were derived from single strains.

**Table 1.** Qualities and putative taxon of each bins in metagenome from N1, N2 and N3 cultures

Bin ID	Putative taxonomy	Complete-ness (%)	Contami-nation (%)	Strain heterog-eneity	Genome size (bp)	No. of contigs	N50	Mean contig length (bp)	Longest contig (bp)	GC	No. of predicted genes
N1-1	* Marinifilum fragile *	99.46	2.15	0	4 632 452	67	126 188	69 141	306 114	35.7	3843
N1-2	* Desulfuromonas* sp.	91.15	2.58	0	4 431 711	306	19 053	14482	125708	55.0	4146
N1-3	* Halodesulfovibrio* sp.	100	0.56	33.33	4 215 690	43	163 138	98039	320254	45.1	3684
N1-4^a^	* Ilyobacter* sp.	94.38	1.12	0	2 867 017	124	32 721	23121	160652	36.3	2715
N1-5	* Prosthecochloris marina *	99.43	1.37	0	2 785 587	24	205628	116066	495280	47.0	2648
N2-1	* Halodesulfovibrio* sp.	97.93	2.73	86.67	3 681 226	182	29684	20226	89738	45.1	3294
N2-2	* Desulfuromonas* sp.	63.38	2.58	40	2 938 736	622	4902	4724	22680	55.8	3037
N2-3^a^	* Ilyobacter* sp.	96.63	1.12	0	2 896 854	127	33041	22809	160613	36.3	2742
N2-4^b^	* Prosthecochloris* sp.	99.45	0.82	0	2 627 088	52	65875	51404	309532	47.4	2545
N2-5	* Desulfovibrio bizertensis *	80.85	1.18	0	2 284 992	440	5709	5193	28696	52.6	2379
N3-1	* Marinifilum* sp.	99.19	2.42	0	5 498 267	61	142436	90135	543023	35.9	4546
N3-2	* Pseudovibrio* sp.	85.04	0.79	0	5 165 768	718	8788	7194	35672	50.0	5091
N3-3	* Halodesulfovibrio* sp.	99.41	0.26	0	3 714 212	81	77081	45854	159330	44.9	3295
N3-4^b^	* Prosthecochloris* sp.	98.90	0.82	0	2 630 645	50	79255	52612	225631	47.4	2545

a, b The bins share >99.95 % ANI with each other

### Novel high-quality CAP draft genomes from coral endolithic cultures

**Fig. 2.**
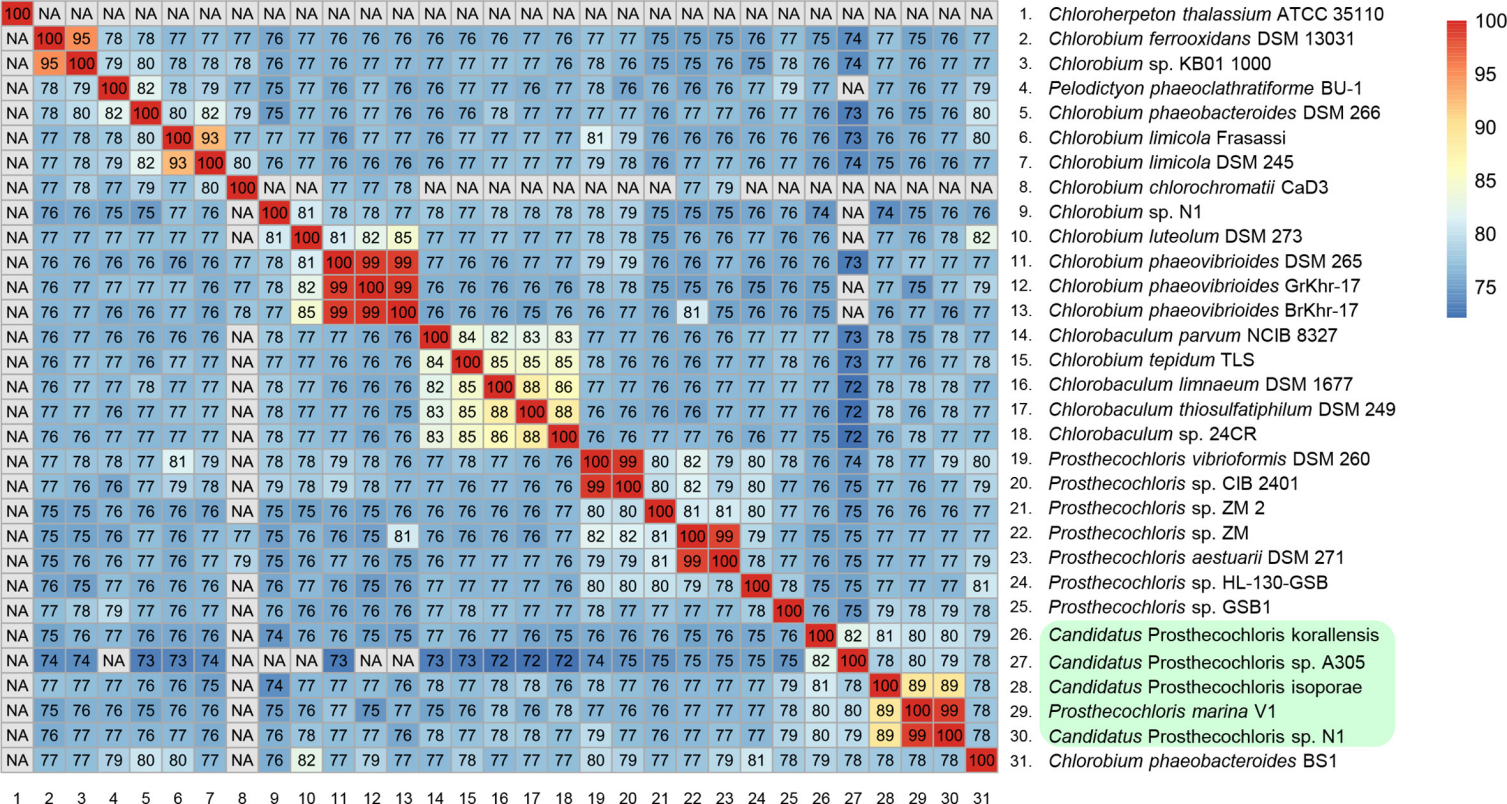
Heatmap of average nucleotide identity between two individual GSB genomes. Values of ANI<70 are denoted as NA because values below 70 % are not reliable. The green shades represent the CAP clade.

The results of the GTDB-Tk taxonomy assignment showed that all *Prosthecochloris *-related bins were closest to *Prosthecochloris marina* V1, which was identified from steel plates in the coastal zone of the South China Sea in 2019 [50]. Interestingly, *Prosthecochloris *-related bins in N2 and N3 shared only 90 % ANI with *Prosthecochloris* marina V1 (Fig. 2), which is below the 95 % ANI cutoff, a frequently used standard for species delineation [51]. On the other hand, the ANI between *Prosthecochloris *-related bins in N2 and N3 was 99.9 %, suggesting that the bins were identical, and these bins were named *Candidatus* Prosthecochloris isoporae. The *Prosthecochloris *-related bins in N2 and N3 was merged by quickmerge [52] to obtain better draft genome assembly, and the merged genome was used as the representative genome for all downstream analysis. The draft genome of *Ca*. P. isoporae was 2.6 Mb with 47.4 % GC, which is within the range of *Prosthecochloris* genomes (2.4–2.7 Mb with 47.0–56.0 % GC). The completeness, contamination, and strain heterogeneity were 99.45, 0.82 and 0 %, respectively. The N50 of the draft genome was 92 kbp. The contig count was 46, and the longest contig was 31.1 kbp.

The ANI between the *Prosthecochloris *-related bin in N1 and *Prosthecochloris marina* V1 was 99 %, suggesting that these genomes belong to the same species. The bin was named *Candidatus* Prosthecochloris sp. N1. Its genome size was 2.7 Mb, with 23 contigs and a 47.0 % GC ratio, which is consistent with the genome of *Prosthecochloris* marina V1 [50].

The ANI between these newly identified genomes and other *Chlorobiaceae* members was also determined (Fig. 2). *Ca*. P. isoporae and *Ca*. P. sp. N1 shared the highest ANI value with *Candidatus* Prosthecochloris sp. A305 (~79 %) and *Candidatus* Prosthecochloris korallensis (~80 %), which were both previously identified from the coral metagenomes and defined as part of the CAP group [11]. Furthermore, the genomes of *Candidatus* Prosthecochloris sp. A305 and *Candidatus* P. korallensis were most similar (82 % ANI) (Fig. 2). These results indicated high genomic similarities between the members of CAP. The other *Chlorobiaceae* closest to CAP were *Prosthecochloris* sp. GSB1 and *Chlorobium phaeobacteroides* BS1, later annotated as *Prosthecochloris phaeobacteroides* BS1 [7].

### Phylogenetic tree of CAP and other green sulfur bacteria

**Fig. 3.**
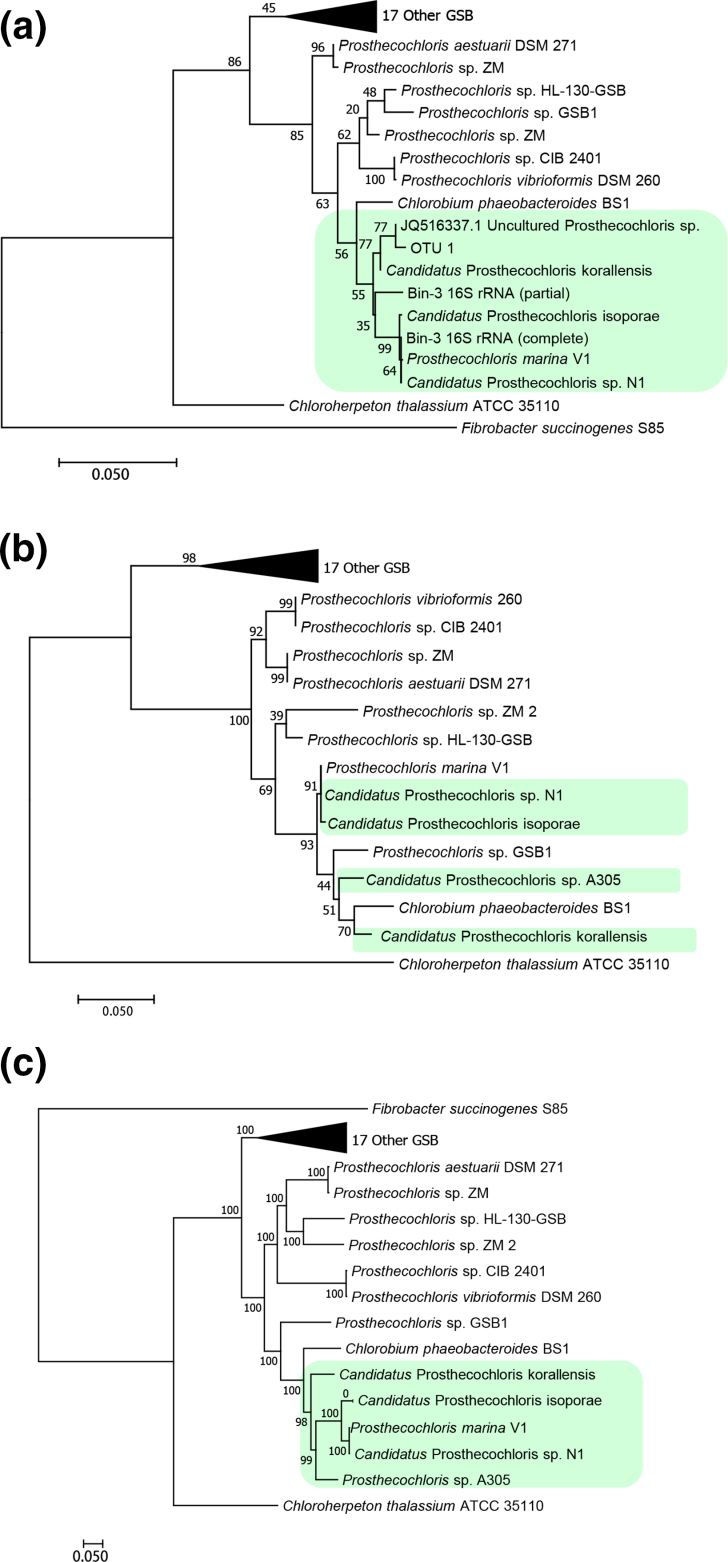
Molecular phylogenetic analysis of green sulphur bacteria. The phylogenetic trees of 16S rRNA (a), FMO protein (b), and 208 concatenated single-copy genes with 75981 amino acid positions (c) were constructed by the maximum-likelihood method with 1000 bootstraps. 27 green sulphur bacteria genomes in the RefSeq database and coral-associated GSB genomes were used to construct the tree. Other GSB included 12 *Chlorobium,* 1 *Pelodictyon* and 4 *Chlorobaculum *. The genome and 16S rRNA sequences of *Fibrobacter succinogenes* S85 were used as the outgroup. The green shades represent the CAP clade.

To determine the phylogenetic relationship between CAP and other members of *Chlorobiaceae *, 16S rRNA gene sequences of CAP-related genomes and other *Chlorobiaceae* were used to reconstruct phylogenetic trees (Fig. 3a). The analysis also included *Prosthecochloris *-related Operational Taxonomic Units (OTU) (at species-like level), which we identified from the green layer of coral *I. palifera* [11]; bin-3, which was recovered from metagenomes in the green layer of *I. palifera* [11]; and one uncultured clone isolated from the coral *Montastraea faveolata* [53]. All CAP members were grouped into the same clade, and the clade closest to it contained other free-living *Prosthecochloris *. The tree based on FMO, a unique photosynthetic-related protein in *Chlorobiaceae *, also classified the CAP members into the same clade, with the addition of *Chlorobium phaeobacteroides* BS1 and *Prosthecochloris* sp. GSB1 (Fig. 3b). In addition, to more confidently establish the evolutionary relationships, we also used concatenated protein sequence alignments of 208 common single-copy genes with 75,981 amino acid positions in these genomes to construct the tree (Fig. 3c). The CAP clade was supported by 98 % bootstrap, which strongly indicated that CAP have a unique evolutionary origin.

### Pan-genome analysis of *Prosthecochloris*

**Fig. 4.**
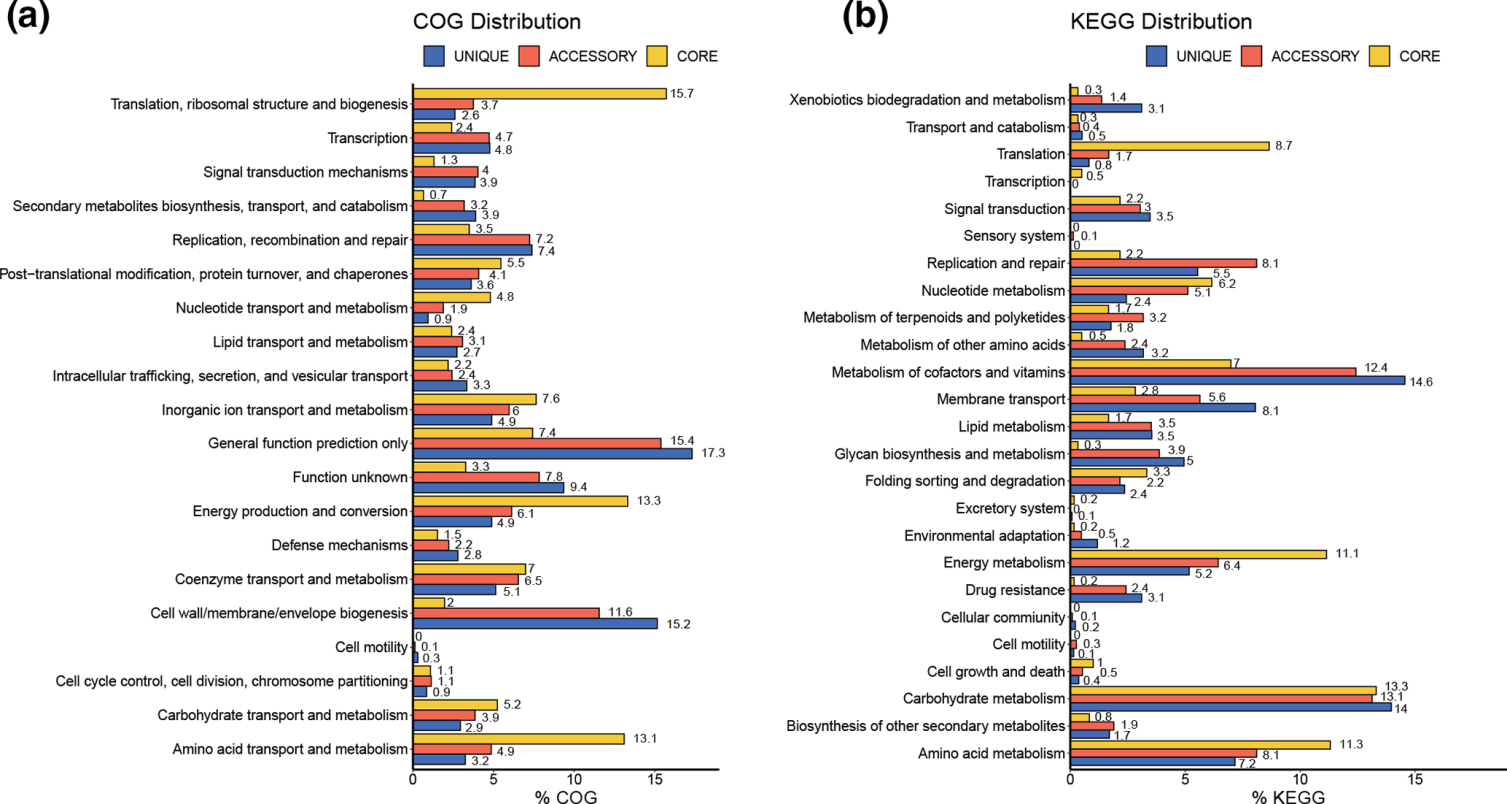
Pan-genome analysis of *Prosthecochloris *. COG (a) and KEGG (b) distributions of core, accessory and unique genes from the pan-genome analysis. *Y*-axes represent the proportions of predicted core, accessory and unique genes in each COG and KEGG functional category.

Pan-genome analysis was conducted to understand the core-accessory relationships in the genus *Prosthecochloris *. The plot of pan-genome size along the number of genomes indicated that the pan-genome is open, indicating that with availability of more sequenced genomes, chances of obtaining new genes is high from *Prosthecochloris *. (Fig. S1a, available in the online version of this article). The *Prosthecochloris* genomes share 442 core genes (Fig. S1b). The number of genes absent only in *Candidatus* Prosthecochloris sp. A305 is 122, which may indicate that the draft genome is incomplete. The COG and KEGG classification of the core, accessory and unique proteins revealed that the translation, energy production and amino acid metabolism categories had higher proportions of core proteins than accessory or unique proteins (Fig. 4a, b). The proportions of core, accessory and unique proteins in translation were 8.7, 1.7 and 0.8%, energy production were 13.3, 6.1 and 4.9%, and amino acid metabolism were 11.3, 8.1 and 7.2%, respectively. On the other hand, the drug resistance, secondary metabolite biosynthesis, DNA replication and membrane transport categories had higher proportions of accessory and unique proteins (Fig. 4a, b). The phylogeny of concatenated alignment of core protein sequences grouped CAP members in the same clade (Fig. S2), with *P*. sp GSB1 and *C. phaeobacteroides* BS1 as closest relatives. The CAP clade contained 213 clade-specific accessory genes. In addition, we also found 80 genes present in all CAP genomes, except that of A305. The 213 accessory genes and these 80 genes were searched using blastn against the NCBI RefSeq database. The results showed that, although most genes had orthologue genes in other *Chlorobiaceae* members, some were unique to CAP members (Table 2). It is noteworthy that the putative gene sources of many blastn top hits were from sulfate-reducing bacteria. Moreover, the d*N/*d*S* ratio of these genes were <0.3, indicating that the changes in amino acid sequences in these gene coding sequences were deleterious.

**Table 2.** Genes present in CAP but absent in other *Chlorobi
*

Query ID	Best hit ID	Description	% identity	A305*	dN/dS†
Org4_Gene107	WP_072283699.1	FMN-binding glutamate synthase family protein [Pelobacter sp. SFB93]	72.3	−	0.094
Org1_Gene1460	WP_084069784.1	TAXI family TRAP transporter solute-binding subunit [Desulfobacterium vacuolatum]	65.1	−	0.15
Org3_Gene1656	WP_027353285.1	TRAP transporter fused permease subunit [Desulfosarcina sp. BuS5]	71.7	−	0.106
Org1_Gene772	WP_047810725.1	GNAT family N-acetyltransferase [Peptococcaceae bacterium CEB3]	60.1	−	0.157
Org3_Gene1412	WP_045900088.1	AadA family aminoglycoside 3′′-O-nucleotidyltransferase [Enterobacter asburiae]	69.8	−	0.246
Org3_Gene1155	WP_093883682.1	DNA-3-methyladenine glycosylase I [Syntrophus gentianae]	69.8	−	0.243
Org1_Gene2204	WP_027367308.1	class I SAM-dependent methyltransferase [Desulfocurvibacter africanus]	69.7	+	0.217
Org1_Gene2431	WP_066061433.1	hypothetical protein (Nuclease?)‡ [*Candidatus* Desulfofervidus auxilii]	67.1	+	0.088
Org1_Gene859	WP_079418068.1	gamma carbonic anhydrase family protein [Thiomonas intermedia]	70.6	+	0.168

*The sign ‘+’ indicates the gene is present in all the CAP genome. ‘–’ represents the gene is present in CAP except for A305.

†The nonsynonymous and synonymous mutation ratio were calculated using the genomes of CAP.

‡The protein function was inferred by TOP 5 hit from blastn.

### Metabolic characteristics of CAP

The KEGG annotation by BlastKoala revealed that all the CAP members have nitrogen fixation genes – except for *Ca*. P. A305 – and lack the genes for dissimilatory nitrate reduction pathway and denitrification – except for *Ca*. P. korallensis, which contains genes responsible for converting nitrite to ammonia (Table S2). For the carbon metabolism pathway, all the CAP members have a complete gene repertoire for the rTCA cycle – except for *Ca*. P. A305, which lacks the *idh* gene. On the other hand, the gene encoding phosphoenolpyruvate carboxylase (*ppc*) is only present in *Ca*. P. A305 and *Ca*. P. korallensis and the carbon monoxide dehydrogenase coding gene (*cooF* or *cooS*) is only present in *Ca*. P. korallensis and *Ca*. P. sp. N1.

For the sulphur metabolism pathways, *sqr* and *fccAB –* encoding sulfide-quinone reductase and sulfide dehydrogenase, respectively – were identified in all CAP members. Complete dissimilatory sulphate reduction (DSR) and thiosulfate reductase pathway encoding genes were identified in all members of CAP except *Ca*. P. A305. In addition, the genomes of *Ca*. P. isoporae and *Ca*. P. sp. N1 also contained all genes in the assimilatory sulphate reduction and thiosulfate-oxidizing Sox enzyme systems, except for the *soxCD* genes.

Distinct colours of the N1 (green) and N2 (brown) cultures led us to hypothesize that CAP can harbour different bacteriochlorophylls (BChl), as a previous study showed that brown-colour GSB have BChl *e* [19]. The KEGG results showed that all CAP members have the genes to synthesize BChl *a*, BChl *b* and BChl *d* from chlorophyllide *a* (Table S2), but the *bciD* gene – encoding the enzyme that converts bacteriochlorophyllide *c* to bacteriochlorophyllide *e –* is only present in *Ca*. P. isoporae. Moreover, our previous analysis of the absorption spectrum revealed the presence of BChl *e* in the N2 culture only [11]. These results implied that the presence of *bciD* gene might enable *Ca*. P. isoporae to synthesize BChl *e*, suggesting that the differences in genes responsible for pigment synthesis could be responsible for the colour difference in the N1 and N2 cultures.

The transporter systems in CAP were also identified by BlastKoala (Table S2). The results demonstrate that CAP have the ABC transporter systems for transporting molybdate, nucleoside, phospholipid, phosphate, lipoprotein, lipopolysaccharide and cobalt. In addition, sulphate, ammonium and drug/metabolite transporters were also identified by annotation in transportDB 2.0.

### Recovered novel sulfate-reducing bacteria genome in coral endolithic cultures

Our binning results showed that the *Halodesulfovibrio *-related bin was present in all coral endolithic cultures, and the bin in N3, bin n3-3, is nearly complete (99.41 %) and has very low contamination (0.26 %) (Table 1). The closest available genome to the bin n3-3 is *Halodesulfovibrio marinisediminis *, with an ANI of 84.1 %, suggesting that the bin belongs to a novel species. Hence, the bin was renamed as *Candidatus* Halodesulfovibrio lyudaonia. The total length of the draft genome is 3.7 Mb, comprising 81 contigs with a 44.9 % GC ratio.

The ANI between the genomes of existing *Halodesulfovibrio* species and *Ca*. H. lyudaonia was 83–84 %. As *Halodesulfovibrio* originally belonged to the *Desulfovibrio* genus, the ANI between *Desulfovibrio* and *Ca*. H. lyudaonia was also determined, which demonstrated that *Ca*. H. lyudaonia and some *Desulfovibrio* species share >70 % ANI. The phylogenetic analysis of 16S rRNA and whole-genome similarity revealed that the *Halodesulfovibrio* could be separated from *Desulfovibrio* as a monophyletic clade (Fig. S3a, b). Besides, the 16S rRNA analysis also showed that *Ca*. H. lyudaonia and *Halodesulfovibrio *-related 16S rRNA in the N1 culture could be classified into a clade with *H. marinisediminis* and *H. spirochaetisodalis* (Fig. S3a).

The genomic analysis within sulphur metabolism revealed that all the existing *Halodesulfovibrio* and *Ca*. H. lyudaonia have dissimilatory sulphate reduction and *sqr* genes (Table S3). For the nitrogen metabolism, the nitrogen-fixation genes were only identified in *H. aestuarii,* and denitrification and nitrate reduction-related genes were absent in all genomes (Table S3). For carbon metabolism, genes participating in glycolysis and ethanol fermentation were present in all *Halodesulfovibrio *. Moreover, all genomes contained multiple genes encoding formate dehydrogenase, which helps convert formate to CO_2_.

The transporter gene analysis revealed the existence of molybdate, nucleoside, phospholipid, phosphate lipopolysaccharide, cobalt, phosphonate, glutamine, branched-amino, zinc and tungstate transporter genes in *Halodesulfovibrio* (Table S3). Furthermore, the general l-amino acid and sulphate transporter genes were also identified in the *Ca*. H. lyudaonia. Different *Halodesulfovibrio* species contained various secretion systems. *Halodesulfovibrio* have genes responsible for the type II secretion system, twin-arginine translocation pathway and general secretory pathway (Table S3). Apart from these systems, the *Ca*. H. lyudaonia also had genes involved in the types III and VI secretion systems.

## Discussion

In this study, we used genomic and functional genomics analyses to characterize CAP and a companion sulfate-reducing bacterium. Two high-quality and high-quality CAP draft genomes were recovered from coral endolithic cultures, including one novel species. The genomic and functional analysis of existing CAP members revealed a functional diversity between the members, in spite of their phylogenetic closeness and genome similarities. Along with CAP, SRB were also common in endolithic cultures, indicating a potential symbiotic relationship between the groups. Hence, a high-quality draft genome of a novel species in *Halodesulfovibrio –* a common SRB genus in coral endolithic cultures – was also recovered and functional genomics analysis performed. Based on the metabolic features of the CAP and SRB genomes, a putative syntrophic interaction between the *Halodesulfovibrio* and CAP was proposed.

### CAP formed a monophyletic clade and shared several CAP-specific genes

*Prosthecochloris* is the only green sulphur bacterial genus found in green layers of coral skeleton to date. Furthermore, CAP can be phylogenetically separated from other free-living *Prosthecochloris *, suggesting that they share certain common features enabling them to live in diverse microenvironments of the coral skeleton. Interestingly, pan-genome analysis identified several genes that were unique to CAP. The similarity search results revealed that most of these genes were from SRB, suggesting a close ecological relationship between SRB and CAP members and maybe even a history of horizontal gene transfer. These CAP-unique genes had a low ratio of nonsynonymous to synonymous substitutions (d*n/*d*s*<1), indicating that these genes underwent purifying selection; therefore, meaning the changes in the overall amino acid sequences of these genes would decrease bacteria fitness.

We propose two hypotheses about the ancestor of CAP. First, it acquired these genes while living in coral skeletons, and these genes were selected for. Second, it lived in other microbial communities and, after acquiring the above mentioned genes, gained fitness to live in coral environments. For example, among the CAP-specific genes, we found that there is a tripartite ATP-independent periplasmic transporter (TRAP transporter) gene cassette that includes permease and a substrate-binding subunit. TRAP is a protein family involved the bidirectional transport of a wide range of organic acids [54]. CAP could potentially use this transport system to acquire important nutrients from the specific coral-built environment.

### CAP possess different photosynthetic machinery

GSB are obligate anaerobic photoautotrophs that use light as an energy source to grow [19]. Photosynthesis occurs in self-assembly light-harvesting complexes called chlorosomes, which comprise different types of bacteriochlorophyll (BChl) pigments [19]. Though all GSB have BChl in their reaction centres, different members have different antenna pigments, resulting in different colours [16]. The major BChls in GSB, including BChl *c*, *d* or *e*, have different absorption peaks. Green-coloured GSB have BChl *c* or *d,* and brown-coloured GSB contain BChl *e* in the chlorosome [16]. The brown-coloured GSB were shown to be well adapted to light-limited environments, such as deeper waters [19]. Moreover, a previous study revealed that light conditions in a lake may determine which colour of GSB will be the dominant group [16, 55].

The coral endolithic cultures N1 and N2, dominated by CAP, were green- and brown-coloured, respectively. Our previous study confirmed the presence of BChl *c* and lack of BChl *e* peak in the N1 culture, from which *Candidatus* P. sp. N1 was recovered [11]. On the other hand, the BChl *e* was present in the N2 culture, from which *Candidatus* P. isoporae was identified. The functional genomics analysis in this study suggests that the lack of the *bciD* gene, which participates in BChl *e* biosynthesis, may account for the absence of BChl *e* in *Ca*. P. sp. N1, leading to the green coloration [56]. This result suggests that CAP members may possess different photosynthetic machinery, which can help species that dominate under different light conditions in coral skeleton microenvironments.

**Fig. 5.**
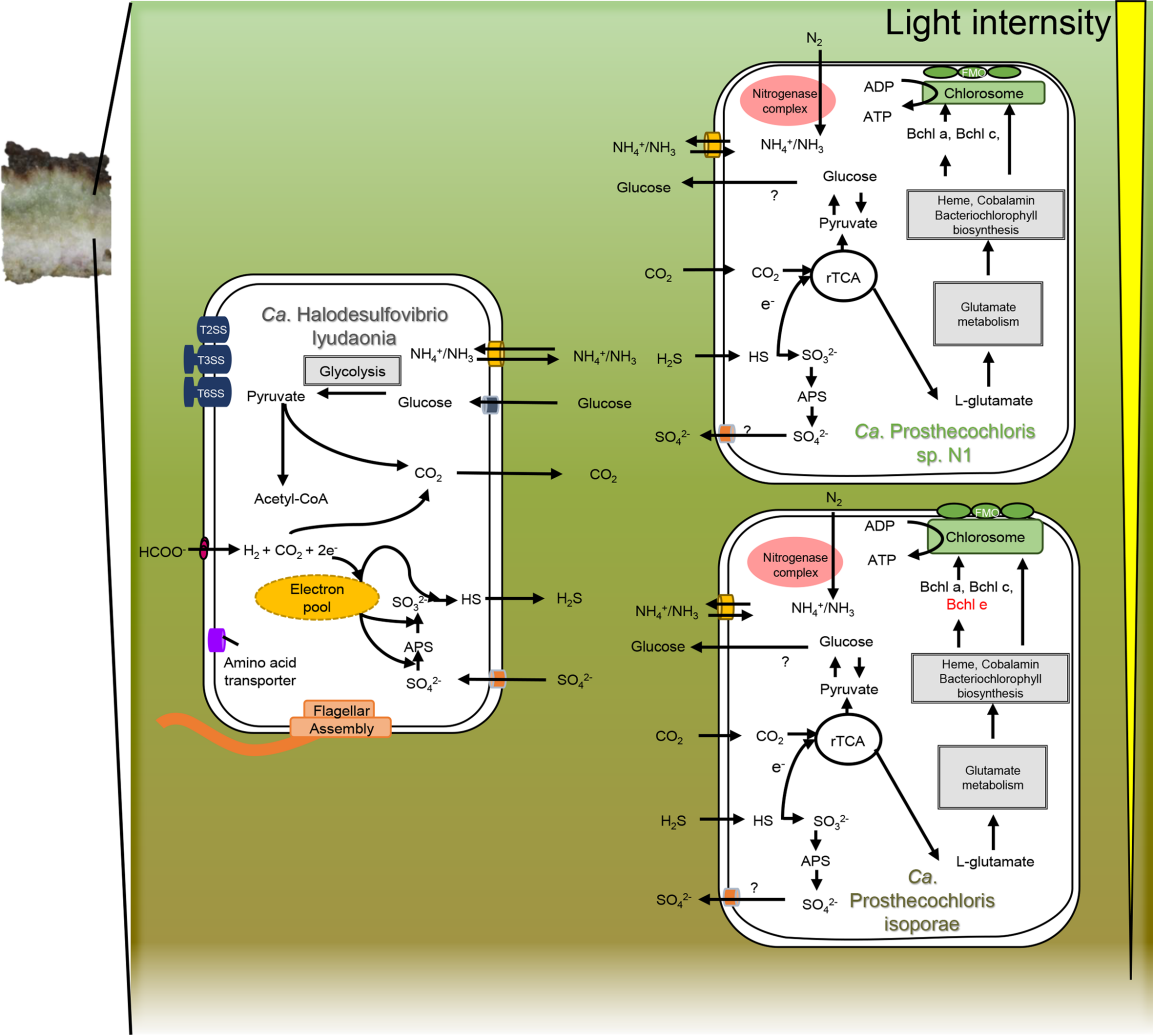
Putative syntrophic interaction between CAP and *Ca*. H. lyudaonia. Brown-coloured *Ca*. P. isoporae dominates the lower section of the coral skeleton while green-coloured *Ca*. P. sp. N1 dominates the upper lower section. The light intensity decreases with depth into the skeleton. The exchange of carbon, sulphur and nitrogen compounds are denoted; important transports are indicated based on the genome annotation. The detailed model is described in the discussion.

Multiple factors contribute to the variation in light availability of a skeleton microenvironment, including individual differences in skeleton pore size and skeleton structures owing to genetic differences or dynamic environmental factors [57]. Light availability also varies at the different depths of the coral tissue [58]. Hence, we hypothesize that the individual difference in skeleton structures and the depth of microhabitat in coral skeleton will influence the distribution of different CAP species. For instance, deeper sections of the skeleton with less light could be dominated by brown-coloured CAP, while the regions closer to the surface of coral tissue may be dominated by green-coloured CAP (Fig. 5). Confirming this hypothesis requires further investigating pigment contents by determining absorbance spectra in the different sections of a single coral skeleton to establish whether there is any correlation between the distribution of the two specific groups and the depth of the skeleton region.

### Sulphur metabolism in CAP

Most GSB species obtain electrons by oxidizing sulfide, sulphur and thiosulfate for carbon fixation [59, 60]. Among oxidative sulphur metabolism pathways, the Sox enzyme system – by which bacteria oxidize thiosulfate – is common. However, using thiosulfate as an electron donor and Sox gene clusters are only found in some GSB [61]. In addition, GSB do not have the SoxCD complex, a part of the Sox system that is integral for oxidizing thiosulfate to sulphate in many other bacteria; instead, the function of SoxCD is replaced by the dissimilatory sulphate reduction (DSR) system in GSB [16, 62, 63]. Moreover, many GSB use the DSR system to oxidize polysulfide to sulfite. Thus, in GSB, the DSR system is required to complete the oxidation of sulphur compounds. In CAP, *Ca*. P. isoporae and *Ca*. P. sp. N1, identified from the coral skeleton, contain all genes involved in DSR and the Sox system – except for *soxCD –* indicating that GSB can obtain electrons by oxidizing sulfide, sulfite and thiosulfate, which is similar to the way that *Chl. tepidum* operates [64]. However, *Ca*. P. korallensis, identified from homogenized corals, only have the DSR system. With the DSR system, GSB are better able to utilize reduced sulphur compounds, which might confer additional advantages in sulfide- and energy-limited conditions. However, *Ca*. P. korallensis lacks the Sox system. This may due to the differences in the availability of sulphur compounds inside corals, which contribute to the diverse sulphur metabolism in CAP or the incompleteness of *Ca*. P. korallensis genome.

In some anaerobic systems, the syntrophic interaction between GSB and SRB occurs because sulphate produced by GSB is used as an electron acceptor in SRB, and biogenic sulfide produced by SRB is used as an electron donor in GSB [20]. The binning results and our previous 16S rRNA gene-based analysis in endolithic cultures revealed the presence of potential SRB including *Halodesulfovibrio *, *Desulfovibrio* and *Desulfuromonas *. These bacteria are common in the skeleton of *I. palifera* [11]. In the three endolithic cultures, the SRB was predominant in metagenomic sequencing, suggesting that it (1) is the main group providing reduced sulphur compounds as electron donors for CAP in cultures and (2) plays the synergetic role in the endolithic community in coral skeletons.

### A novel sulfate-reducing bacterium genome identified from coral endolithic cultures

Our metagenome analyses demonstrated the relationship between CAP and SRB. The most abundant SRB in our coral endolithic cultures is *Halodesulfovibrio *, which is present in all cultures and also in green layers. Here, we recovered a high-quality draft genome of a novel species *Candidatus* Halodesulfovibrio lyudaonia. *Halodesulfovibrio* was classified as a novel genus separated from *Desulfovibrio* according to the differences in genome, phylogeny and phenotype in 2017 [65–67]. There are currently only four available species and genomes, which were all identified from marine habitats, including sediment and oxygen minimum zone water columns. Ours is the first study to find that *Halodesulfovibrio* might have a relationship with its eukaryotic host and may have syntrophic relationship with other bacteria.

Previous studies revealed that *Halodesulfovibrio* can use sulphate or sulfite as electron acceptors [67]. The presence of all genes involved in the DSR system indicates that these bacteria use this pathway to reduce sulphur compounds (Table S3). In addition, some SRB can also fix nitrogen, such as *Firmicutes* and *Deltaproteobacteria* [68]. In our analysis, nitrogen fixation genes were absent in all *Halodesulfovibrio* except *H. aestuarii* (Table S3). However, we also found that bacteria containing the gene encoding l-amino acid and ammonia transporters can be used to obtain organic nitrogen.

### Putative syntrophic interaction between diverse CAP and *Halodesulfovibrio*

Previously, we proposed a general syntrophic interaction based on a gene-centric approach with metagenomes of coral skeleton [11]. Here, using several high-quality draft genomes from endolithic cultures, we identified CAP and SRB species that participate in this syntrophic interaction. Moreover, the high-quality draft genomes also allowed us to characterize communities and interactions in a more accurate and detailed manner. The recovered genomes highlight the diversity in CAP and the complex interactions in the community (Fig. 5).

Brown-coloured CAP can adapt to low-light microenvironments, and therefore may dominate deeper sections of the skeleton, while green-coloured CAP may dominate the sections closer to the coral tissue, which are exposed to relatively higher light intensity. On the other hand, the presence of *Halodesulfovibrio* in all endolithic cultures – along with both brown- and green-coloured CAP – suggests that *Halodesulfovibrio* may be distributed across different sections and interact with both colours of CAP. We suggest that both CAP species occupy their niches via diversified pigment compositions, and both interact in a syntrophic manner with *Halodesulfovibrio *.

During photosynthesis, these CAP obtain CO_2_ released by *Halodesulfovibrio* and other heterotrophs. To fix carbon through the rTCA cycle, CAP obtains sulfide from *Halodesulfovibrio* as an electron donor*,* while the *Halodesulfovibrio* obtain oxidized sulphur compounds released from CAP and reduce them using electrons from the conversion of formate to CO_2_. Therefore, CAP and *Halodesulfovibrio* provide each other with sulphur resources in the coral skeleton.

Being the most dominant nitrogen fixers, CAP fixes dinitrogen into ammonium, which can be bi-directionally diffused across the cell membrane into the microenvironment by the ammonium transporter. Although genes involved in nitrogen fixation are absent in *Halodesulfovibrio *, they can take up ammonium through an ammonium transporter, which might serve as a potential nitrogen source. Hence, we suggest that CAP plays an essential role in nitrogen fixation in the community.

## Conclusion

Though the skeleton microbiome may contain nutritional sources and facilitate the recovery of unhealthy coral [15], its importance in the coral skeleton has been overlooked, and the interactions inside the community are poorly studied due to methodological limitations [21]. Here, our genomic analysis of endolithic cultures helps us better characterize the community and investigate the interaction between coral and the endolithic microbiome.

Endolithic cultures provide several high-quality and precise genomes to study endolithic communities. Genomic analysis revealed that members of CAP share a common origin and contain several CAP-specific genes, indicating that certain differences exist between CAP and other free-living *Prosthecochloris *. These differences imply that coral and CAP have a symbiotic relationship, but future investigations into metabolic exchanges between CAP and the coral host are needed to confirm this. On the other hand, functional genomic analysis revealed the diversity of pigments synthesized in CAP, suggesting that (1) individual members of CAP adapt to different microenvironments in the skeleton and (2) there is spatial heterogeneity in the microbiome. Along with CAP, the predominance of *Halodesulfovibrio* indicates that it is ecologically important in skeleton microbiome communities. Based on their metabolic features, we characterize the carbon, sulphur, nitrogen cycling between *Halodesulfovibrio* and CAP, specifying the metabolic relationships among endolithic microbes in corals.

## Supplementary Data

Supplementary material 1
