# Supplementary material for: Potential syntrophic relationship between coral-associated Prosthecochloris and its companion sulfate-reducing bacterium unveiled by genomic analysis
Source: Microb Genom. 2021 May 5;7(5):000574. doi: 10.1099/mgen.0.000574 (PMC8209720; doi:10.1099/mgen.0.000574)
Supplement: Supplementary material 1 [file mgen-7-0574-s001.pdf]

Supplementary Data

Potential syntrophic relationship between coral-associated Prosthecochloris and its companion sulfate-reducing bacterium unveiled by genomic analysis

Yu-Hsiang Chen[1,2,3*], Shan-Hua Yang[4*], Kshitij Tandon[2,3,5], Chih-Ying Lu[3,6,7], Hsing-Ju Chen[3], Chao-Jen Shih[8], Sen-Lin Tang[2,3,6]#

[1]Bioinformatics Program, Taiwan International Graduate Program, National Taiwan University, Taipei, Taiwan

[2]Bioinformatics Program, Institute of Information Science, Taiwan International Graduate Program, Academia Sinica, Taipei, Taiwan

[3]Biodiversity Research Center, Academia Sinica, Taipei, Taiwan

[4]Institute of Fisheries Science, National Taiwan University, Taipei, Taiwan

[5]Institute of Molecular and Cellular Biology, National Tsing Hua University, Hsinchu, Taiwan

[6]Molecular and Biological Agricultural Sciences Program, Taiwan International Graduate Program, National Chung Hsing University and Academia Sinica, Taipei, Taiwan

[7]Graduate Institute of Biotechnology, National Chung Hsing University, Taichung, Taiwan

[8]Bioresource Collection and Research Center, Food Industry Research and Development Institute, Hsinchu, Taiwan

*Yu-Hsiang Chen and Shan-Hua Yang contributed equally to this work.

# Corresponding author email: sltang@gate.sinica.edu.tw

Supplementary Figures

a

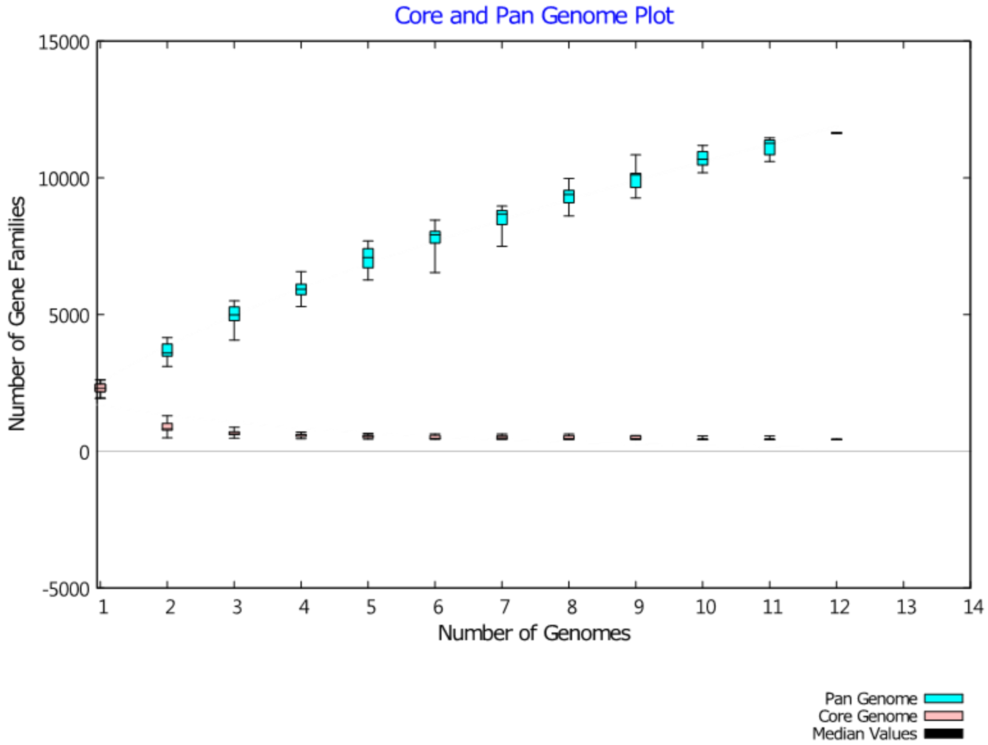

b

Organism name	No. of core genes	No. of accessory genes	No. of unique genes	No. of exclusively absent genes
Candidatus Prosthecochloris korallensis	443	1448	574	2
Candidatus Prosthecochloris sp. A305	443	1085	414	123
Candidatus Prosthecochloris sp. N1	443	1812	354	1
Candidatus Prosthecochloris isoporaea	443	1731	338	2
Chlorobium phaeobacteroides BS1	443	1230	796	1
Prosthecochloris sp. GSB1	443	797	1049	2
Prosthecochloris aestuarii DSM 271	443	1651	228	0
Prosthecochloris sp. CIB 2401	443	1466	252	0
Prosthecochloris sp. HL-130-GSB	443	904	956	4
Prosthecochloris sp. ZM 2	443	911	842	1
Prosthecochloris sp. ZM	443	1719	269	0
Prosthecochloris vibrioformis DSM_260	443	1490	179	0

FIG S1. Pan-genome analysis. (a) Core and Pan-Genome plot of *Prosthecochloris*. (b) Statistics from the pan-genome analysis, including number of core, unique, accessory, and exclusive absent genes. The green shades represent the CAP clade.

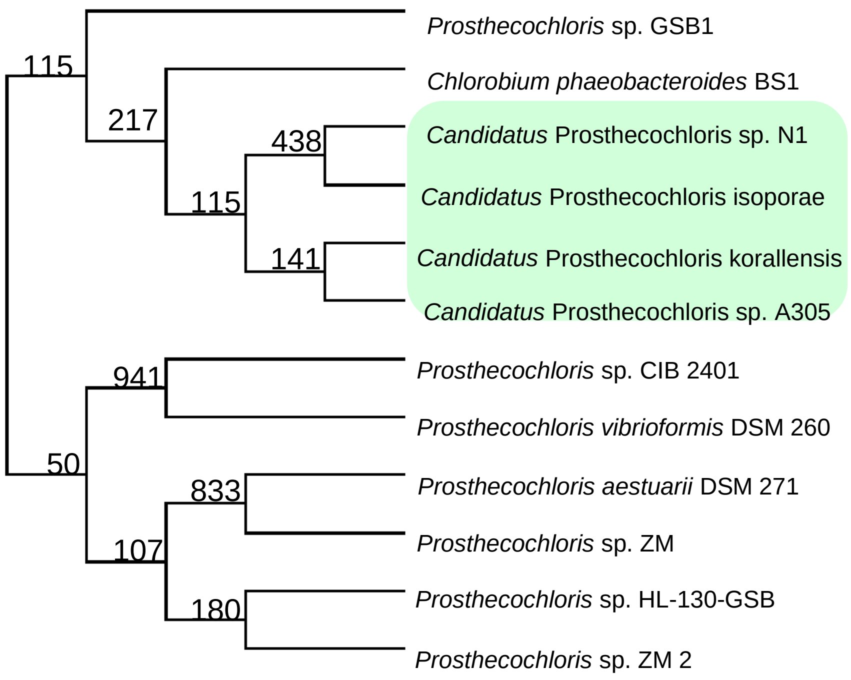

FIG S2. Phylogenetic tree based on core genome. The protein sequences of 20 random orthologous gene clusters in the core genome were aligned by MUSCLE, and the tree were constructed and concatenated by the neighbor-joining method. The number of clade-specific accessory genes are shown in each branch. The green shades represent the CAP clade.

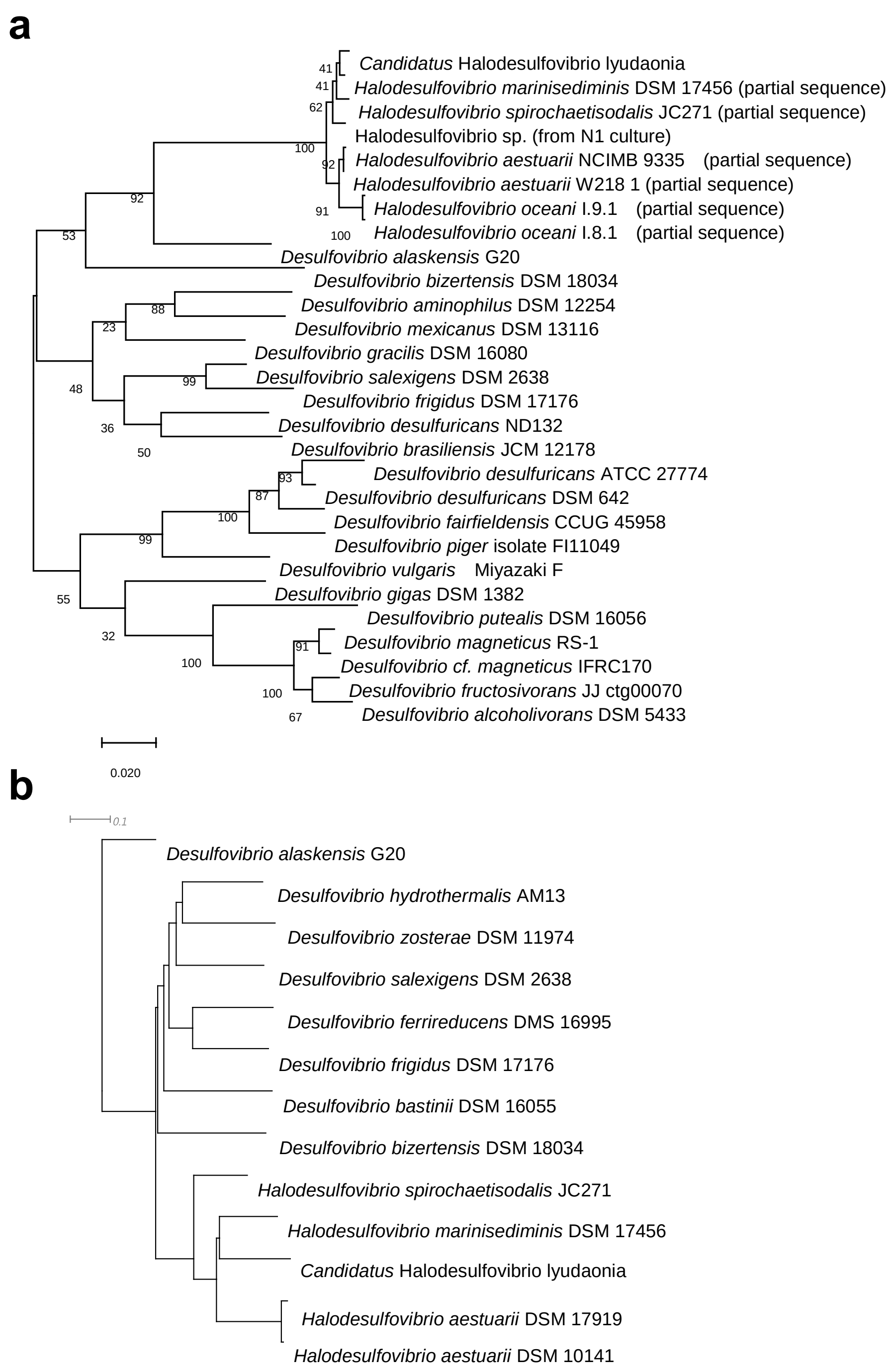

FIG S3. Molecular phylogenetic analysis of *Desulfovibrio* (a) Phylogeny constructed from 16S rRNA from 20 *Desulfovibrio* genomes from the RefSeq database and *Halodesulfovibrio* using the maximum-likelihood method with 1000 bootstraps. 28 sequences and 1562 position were involved in the analysis. Scare bar represents 0.02 changes per nucleotide site. (b) Similarity matrix between each of the two SRB genomes created by Gegenees. The matrix was exported into a distance matrix, which was used to generate a dendrogram by SplitsTree 4 with the neighbor joining method. The scare bar indicates 1% difference among average BLASTN similarity scores.

Table S1. Mapped reads and inferred abundance of each bin in N1, N2, and N3 metagenomes.

Bin ID	putative taxon	Bin size (Mbp)	mapped reads	% mapped reads	% binned populations	% community
N1-1	*Marinifilum fragile*	4.63	279,938	5.34	3.73	3.48
N1-2	*Desulfuromonas* sp.	4.43	181,095	3.46	2.52	2.35
N1-3	*Halodesulfovibrio* sp.	4.22	389,110	7.42	5.70	5.32
N1-4	*Ilyobacter* sp.	2.87	2,524,029	48.16	54.34	50.73
N1-5	*Prosthecochloris marina*	2.78	1,518,759	28.98	33.72	31.48
Unbinned		11.05	347,869	6.64	NA	6.64
N2-1	*Halodesulfovibrio* sp.	3.68	187,458	4.13	3.37	3.05
N2-2	*Desulfuromonas* sp.	2.94	77,864	1.72	1.75	1.59
N2-3	*Ilyobacter* sp.	2.90	1,005,127	22.15	22.94	20.79
N2-4	*Prosthecochloris* sp.	2.62	2,780,132	61.26	70.11	63.56
N2-5	*Desulfovibrio bizertensis*	2.28	63,396	1.40	1.83	1.66
Unbinned		14.01	424,156	9.35	NA	9.35
N3-1	*Marinifilum* sp.	5.50	401,910	4.21	2.28	2.10
N3-2	*Pseudovibrio* sp.	5.17	178,820	1.87	1.08	0.99
N3-3	*Halodesulfovibrio* sp.	3.71	230,688	2.42	1.93	1.78
N3-4	*Prosthecochloris* sp.	2.63	7,987,847	83.66	94.71	87.29
unbinned		13.21	748,174	7.84	NA	7.84

Table S2. Metabolism pathways and ABC transporter in coral-associated *Prosthecochloris*.

Sulfur metabolism

Category	Reaction	Gene	*Ca*. P. korallensis	*Ca*. P. sp. A305	*Ca*. P. sp. N1	*Ca*. P. isoporae	*P. marina* V1
Assimilatory sulfate reduction	Sulfate-->APS	*papss*	.	.	.	.	.
Assimilatory sulfate reduction	Sulfate-->APS	*sat*	+	.	+	+	+
Assimilatory sulfate reduction	Sulfate-->APS	*cysND*	.	.	+	+	+
Assimilatory sulfate reduction	APS-->PAPS	*cysC*	.	.	+	+	+
Assimilatory sulfate reduction	PAPS-->Sulfite	*cysH*	.	.	.	.	.
Dissimilatory sulfate reduction and oxidation	Sulfate-->APS	*sat*	+	.	+	+	+
Dissimilatory sulfate reduction and oxidation	APS-->Sulfite	*aprAB*	+	.	+	+	+
Dissimilatory sulfate reduction and oxidation	Sulfite-->Sulfide	*dsrAB*	+	.	+	+	+
SOX system	Thiosulfate-->SoxYZ-S-SSO3	*soxA*	.	.	+	+	+
SOX system	Thiosulfate-->SoxYZ-S-SSO3	*soxX*	.	.	+	+	+
SOX system	SoxYZ-S-S-SO3-->Sulfate	*soxB*	.	.	+	+	+
SOX system	SoxYZ-S-S-SO3-->SoxYZ-S-SH	*soxB*	.	.	+	+	+
SOX system	SoxYZ-S-SH-->SoxYZ-SH	*soxY*	.	.	+	+	+
SOX system	SoxYZ-S-SH-->SoxYZ-SH	*soxZ*	.	.	+	+	+
SOX system	SoxYZ-S-SH-->SoxYZ-S-SO3	*soxC*	.	.	.	.	.
SOX system	SoxYZ-S-SH-->SoxYZ-S-SO3	*soxD*	.	.	.	.	.
SOX system	SoxYZ-S-SO3-->SoxYZ-SH	*soxB*	.	.	+	+	+
Other	Thiosulfate-->Sulfide	*phsA,psrA, phsB,phsC*	+	.	+	+	+
Other	Sulfide-->(Sulfide)n-1	*sqr*	+	+	+	+	+
Other	Sulfide-->Sulphur	*fccB,fccA*	+	+	+	+	+

Carbom metabolism

Category	Reaction	Gene	*Ca*. P. korallensis	*Ca*. P. sp. A305	*Ca*. P. sp. N1	*Ca*. P. isoporae	*P. marina* V1
Reductive Citric acid cycle	Acetyl-CoA-->Pyruvate	*por*	+	+	+	+	+
Reductive Citric acid cycle	Pyruvate-->Phosphoenol-pyruvate	*ppdK*	+	+	+	+	+
Reductive Citric acid cycle	CO2+Phosphoenol-pyruvate->Oxaloacetate	*ppc*	+	+	.	.	.
Reductive Citric acid cycle	Oxaloacetate-->Malate	*mdh*	+	+	+	+	+
Reductive Citric acid cycle	Malate-->Fumarate	*fum*	+	+	+	+	+
Reductive Citric acid cycle	Fumarate -->Succinate	*kor*	+	+	+	+	+
Reductive Citric acid cycle	Succinate-->Succinyl-CoA	*suc*	+	+	+	+	+
Reductive Citric acid cycle	Succinyl-CoA+CO2-->2-Oxogluarate	*kor*	+	+	+	+	+
Reductive Citric acid cycle	2-Oxogluarate+CO2-->Isocitrate	*idh*	+	.	+	+	+
Reductive Citric acid cycle	Isocitrate-->cis-Aconitate	*aco,acnB*	+	+	+	+	+
Reductive Citric acid cycle	cis-Aconitate-->Citrate	*ACO,acnB*	+	+	+	+	+
Reductive Citric acid cycle	Citrate-->Acetyl-CoA	*ACLY,aclA,aclA*	+	.	+	+	+
Other	CO2-->CO	*cooS,cooF*	+	.	+	.	+

The sign '+' indicates the gene is present in the genome.

Nitrogen Metabolism

Category	Reaction	Gene	*Ca*. P. korallensis	*Ca*. P. sp. A305	*Ca*. P. sp. N1	*Ca*. P. isoporae	*P. Marina* V1
Dissimilatory nitrate reduction	Nitrite-->Ammonia	*NirBD*	+	.	.	.	.
Dissimilatory nitrate reduction	Nitrite-->Ammonia	*NrfAH*	.	.	.	.	.
Denitrification	Nitrate-->Nitrite	*NarGHI,NapAB*	.	.	.	.	.
N2 fixation	Nitrogen-->Ammonia	*NifDKH*	+	.	+	+	+
Other	Ammonia-->L-Glutamine	*GLUL,glnA*	.	.	+	+	+
Other	CO2-->HCO3	*Caronic Anhydrase*	+	+	+	+	+
Other	Hydroxylamine-->Ammonia	*hcp+nirS*	+	+	+	+	+

Bacteriochlorophyll Synthesis

Step	Reaction	Gene	*Ca*. P. korallensis	*Ca*. P. sp. A305	*Ca*. P. sp. N1	*Ca*. P. isoporae	*P. Marina* V1
bcl a, b synthesis	Chlorophyllide a-->3-Hydroxyethyl-bacteriochlorophyllide a	*bchX,bchY,bchZ*	+	+	+	+	+
bcl a, b synthesis	Chlorophyllide a-->3-Hydroxyethyl-bacteriochlorophyllide a	*bchF*	+	.	+	+	+
bcl a, b synthesis	3-Hydroxyethyl-bacteriochlorophyllide a-->bacteriochlorophyllide a	*bchC*	+	.	+	+	+
bcl a, b synthesis	bacteriochlorophyllide a --> Geranylgeranyl bacteriochlorophyll a	*chlG*	+	+	+	+	+
bcl a, b synthesis	Geranylgeranyl bacteriochlorophyll a--> Bacteriochlorophyll a	*chlP*	+	+	+	+	+
bcl c ,d, e synthesis	Chlorophyllide a --> 8-Ethyl-12-methyl-3-vinylbacteriochlorophyllide	*bciC*	+	+	+	+	+
	8-Ethyl-12-methyl-3-vinylbacteriochlorophyllide ->bacteriochlorophyllide d	*bchV*	*	*	*	*	*
bcl c ,d, e synthesis	Bacteriochlorophyllide d --> Bacteriochlorophyllide c	*bchU*	+	+	+	+	+
bcl c ,d, e synthesis	Bacteriochlorophyllide d --> Bacteriochlorophyll d	*bchK*	*	*	*	*	*
bcl c ,d, e synthesis	Bacteriochlorophyllide c --> Bacteriochlorophyll c	*bchK*	*	*	*	*	*
bcl e synthesis	Bacteriochlorophyllide c --> Bacteriochlorophyllide e	*bciD*	.	.	.	+	.

ABC Transporter

transporter system	Reaction	Gene	*Ca*. P. korallensis	*Ca*. P. sp. A305	*Ca*. P. sp. N1	*Ca*. P. isoporaea	*P. Marina* V1
Molybate	substrate-binding protein	*modA*	+	+	+	+	+
Molybate	permease protein	*modB*	+	+	+	+	+
Molybate	ATP-binding protein	*modC*	+	+	+	+	+
Molybate	ATP-binding protein	*modF*	.	.	.	.	.
Nucleoside	basic membrane protein A	*bmpA*	+	+	+	+	+
Nucleoside	permease protein	*nupC*	+	+	+	+	+
Nucleoside	permease protein	*nupB*	+	+	+	+	+
Nucleoside	ATP-binding protein	*nupA*	+	+	+	+	+
Phospholipid	substrate-binding protein	*mlaC*	.	.	.	.	.
Phospholipid	substrate-binding protein	*mlaD*	+	.	+	+	+
Phospholipid	permease protein	*mlaE*	+	.	+	+	+
Phospholipid	ATP-binding protein	*mlaF*	+	.	+	+	+
Phospholipid	ATP-binding protein	*mlaB*	.	.	.	.	.
Phosphate	substrate-binding protein	*PstS*	+	+	+	+	+
Phosphate	permease protein	*PstC*	+	+	+	+	+
Phosphate	permease protein	*PstA*	+	+	+	+	+
Phosphate	ATP-binding protein	*PstB*	+	+	+	+	+
Lipoprotein	permease protein	*lolC_E*	+	+	+	+	+
Lipoprotein	ATP-binding protein	*lolD*	+	+	+	+	+
Lipopolysaccharide	permease protein	*lptF*	+	+	+	+	+
Lipopolysaccharide	ATP-binding protein	*lptB*	+	+	+	+	+
Lipopolysaccharide	permease protein	*lptG*	+	.	+	+	+
Cobalt / Nickel	permease protein	*CbiN*	+	+	+	+	+
Cobalt / Nickel	permease protein	*CbiM*	+	+	+	+	+
Cobalt / Nickel	permease protein	*CbiQ*	+	+	+	+	+
Cobalt / Nickel	ATP-binding protein	*CbiO*	+	+	+	+	+

The sign '+' indicates the gene is present in the genome.

Table S3. Metabolism pathways and transporter systems in *Halodesulfovibrio*.

Sulfur metabolism

Category	Reaction	Gene	*Ca.* H. lyudaonia	*H. aestuarii* DSM 10141	*H. aestuarii* DSM 17919	*H. marinisediminis* DSM 17456	*H. spirochaetisodalis* JC271
Assimilatory sulfate reduction	Sulfate-->APS	papss	.	.	.	.	.
Assimilatory sulfate reduction	Sulfate-->APS	Sat	+	+	+	+	+
Assimilatory sulfate reduction	Sulfate-->APS	cysND	.	.	.	.	.
Assimilatory sulfate reduction	APS-->PAPS	cysC	.	.	.	.	.
Assimilatory sulfate reduction	PAPS-->Sulfite	cysH	+	+	+	+	+
Dissimilatory sulfate reduction	Sulfate-->APS	sat	+	+	+	+	+
Dissimilatory sulfate reduction	APS-->Sulfite	aprAB	+	+	+	+	+
Dissimilatory sulfate reduction	Sulfite-->Sulfide	dsrAB	+	+	+	+	+
SOX system	Thiosulfate-->SoxYZ-S-SSO3	soxA	.	.	.	.	.
SOX system	Thiosulfate-->SoxYZ-S-SSO3	soxX	.	.	.	.	.
SOX system	SoxYZ-S-S-SO3-->Sulfate	soxB	.	.	.	.	.
SOX system	SoxYZ-S-S-SO3-->SoxYZ-S-SH	soxB	.	.	.	.	.
SOX system	SoxYZ-S-SH-->SoxYZ-SH	soxY	.	.	.	.	.
SOX system	SoxYZ-S-SH-->SoxYZ-SH	soxZ	.	.	.	.	.
SOX system	SoxYZ-S-SH-->SoxYZ-S-SO3	soxC	.	.	.	.	.
SOX system	SoxYZ-S-SH-->SoxYZ-S-SO3	soxD	.	.	.	.	.
SOX system	SoxYZ-S-SO3-->SoxYZ-SH	soxB	.	.	.	.	.
Other	Thiosulfate-->Sulfide	phsA,psrA, phsB,phsC	.	+	+	.	.
Other	Sulfide-->(Sulfide)n-1	sqr	+	+	+	+	+
Other	Sulfide-->Sulphur	fccB,fccA	.	.	.	.	.

Nitrogen metabolism

Category	Reaction	Gene	*Ca.* H. lyudaonia	*H. aestuarii* DSM 10141	*H. aestuarii* DSM 17919	*H. marinisediminis* DSM 17456	*H. spirochaetisodalis* JC271
Dissimilatory nitrate reduction	Nitrite-->Ammonia	nirBD	.	.	.	.	.
Dissimilatory nitrate reduction	Nitrite-->Ammonia	nrfAH	.	.	.	.	.
Denitrification	Nitrate-->Nitrite	narGHI,napAB	.	.	.	.	.
N2 fixation	Nitrogen-->Ammonia	nifDKH	.	+	+	.	.
Other	Ammonia-->L-Glutamine	GLUL,glnA	+	+	+	+	+
Other	CO2-->HCO3	Caronic Anhydrase	+	+	+	+	+
Other	Hydroxylamine-->Ammonia	hcp+nirS	+	+	+	+	+

The sign '**+**' indicates the gene is present in the genome.

Carbon metabolism

Category	Reaction	Gene	*Ca.* H. lyudaonia	*H. aestuarii* DSM 10141	*H. aestuarii* DSM 17919	*H. marinisediminis* DSM 17456	*H. spirochaetisodalis* JC271
Glycolysis	beta-D-Glucose <=> alpha-D-Glucose	*galM*	+	+	+	+	+
Glycolysis	ATP + alpha-D-Glucose <=> ADP + alpha-D-Glucose 6-phosphate	*GCK*	+	+	+	+	+
Glycolysis	alpha-D-Glucose 6-phosphate <=> beta-D-Fructose 6-phosphate	*GPI, pgi, pgi1, tal-pgi, pgi-pmi*	+	+	+	+	+
Glycolysis	ATP + beta-D-Fructose 6-phosphate <=> ADP + beta-D-Fructose 1,6-bisphosphate	*pfkA, PFK, pfkB, pfk, pfp*	+	+	+	+	+
Glycolysis	beta-D-Fructose 1,6-bisphosphate <=> Glycerone phosphate + D-Glyceraldehyde 3-phosphate	*ALDO, FBA, fbaA, fbaB*	+	+	+	+	+
Glycolysis	D-Glyceraldehyde 3-phosphate + Orthophosphate + NAD+ <=> 3-Phospho-D-glyceroyl phosphate + NADH + H+	*GAPDH, gapA*	+	+	+	+	+
Glycolysis	ADP + 3-Phospho-D-glyceroyl phosphate <=> ATP + 3-Phospho-D-glycerate	*pgk*	+	+	+	+	+
Glycolysis	3-Phospho-D-glycerate <=> 2-Phospho-D-glycerate	*gpmI, gpmB, apgM*	+	+	+	+	+
Glycolysis	2-Phospho-D-glycerate <=> Phosphoenolpyruvate + H2O	*ENO, eno*	+	+	+	+	+
Glycolysis	ADP + Phosphoenolpyruvate <=> ATP + Pyruvate	*PK, pyk, PKLR*	+	+	+	+	+
Glycolysis	alpha-D-Glucose <=> beta-D-Glucose	*galM, GALM*	+	+	+	+	+
Glycolysis	ATP + alpha-D-Glucose <=> ADP + alpha-D-Glucose 6-phosphate	*GCK*	+	+	+	+	+
Other	Ethanol + NAD+ <=> Acetaldehyde + NADH + H+	*ADH*	+	+	+	+	+
Other	Ethanol + NADP+ <=> Acetaldehyde + NADPH + H+	*AKR1A1, adh*	+	+	+	+	+
Other	formate dehydrogenase ; formate <=> CO2	*FDH, fdoG, fdhF, fdwA, fdwB, fdoH, fdsB, fdoI, fdsG, fdsD, fdhA, fdhB*	+	+	+	+	+

ABC trasnporters

transporter system	Reaction	Gene	*Ca.* H. lyudaonia	*H. aestuarii* DSM 10141	*H. aestuarii* DSM 17919	*H. marinisediminis* DSM 17456	*H. spirochaetisodalis* JC271
Molybate	substrate-binding protein	*modA*	+	+	+	+	+
Molybate	permease protein	*modB*	+	+	+	+	+
Molybate	ATP-binding protein	*modC*	+	+	+	+	+
Molybate	ATP-binding protein	*modF*	+	+	+	.	+
Ion(III)	substrate-binding protein	*afuA*	+	.	.	+	.
Ion(III)	permease protein	*afuB*	+	.	.	+	.
Ion(III)	ATP-binding protein	*afuC*	+	.	.	+	.
Nucleoside	basic membrane protein A	*bmpA*	+	+	+	+	+
Nucleoside	permease protein	*nupC*	+	+	+	+	+
Nucleoside	permease protein	*nupB*	+	+	+	+	+
Nucleoside	ATP-binding protein	*nupA*	+	+	+	+	+
Phospholipid	substrate-binding protein	*mlaC*	+	+	+	+	+
Phospholipid	substrate-binding protein	*mlaD*	+	+	+	+	+
Phospholipid	permease protein	*mlaE*	+	+	+	+	+
Phospholipid	ATP-binding protein	*mlaF*	+	+	+	+	+
Phospholipid	ATP-binding protein	*mlaB*	+	+	+	+	+
Phosphate	substrate-binding protein	*pstS*	+	+	+	+	+
Phosphate	permease protein	*pstC*	+	+	+	+	+
Phosphate	permease protein	*pstA*	+	+	+	+	+
Phosphate	ATP-binding protein	*pstB*	+	+	+	+	+
Lipoprotein	permease protein	*lolC_E*	+	+	+	+	+
Lipoprotein	ATP-binding protein	*lolD*	+	+	+	+	+
Lipopolysaccharide	permease protein	*lptF*	+	+	+	+	+
Lipopolysaccharide	ATP-binding protein	*lptB*	+	+	+	+	+
Lipopolysaccharide	permease protein	*lptG*	+	+	+	+	+
Cobalt / Nickel	permease protein	*cbiN*	+	+	+	+	+
Cobalt / Nickel	permease protein	*cbiM*	+	+	+	+	+
Cobalt / Nickel	permease protein	*cbiQ*	+	+	+	+	+
Cobalt / Nickel	ATP-binding protein	*cbiO*	+	+	+	+	+

ABC trasnporters

transporter system	Reaction	Gene	*Ca.* H. lyudaonia	*H. aestuarii* DSM 10141	*H. aestuarii* DSM 17919	*H. marinisediminis* DSM 17456	*H. spirochaetisodalis* JC271
phosphonate	substrate-binding protein	*phnD*	+	+	+	+	+
phosphonate	permease protein	*phnE*	+	+	+	+	+
phosphonate	ATP-binding protein	*phnC*	+	+	+	+	+
glutamine	substrate-binding protein	*glnH*	+	+	+	+	+
glutamine	permease protein	*glnP*	+	+	+	+	+
glutamine	ATP-binding protein	*glnQ*	+	+	+	+	+
general L-amino acid	substrate-binding protein	*aapJ, bztA*	+	+	+	+	+
general L-amino acid	permease protein	*aapQ, bztB*	+	.	.	+	+
general L-amino acid	permease protein	*aapM, bztC*	+	.	.	+	+
general L-amino acid	ATP-binding protein	*aapP, bztD*	+	+	+	+	+
Branched-amino acid	substrate-binding protein	*livK*	+	+	+	+	+
Branched-amino acid	permease protein	*livH*	+	+	+	+	+
Branched-amino acid	permease protein	*livM*	+	+	+	+	+
Branched-amino acid	ATP-binding protein	*livG*	+	+	+	+	+
Branched-amino acid	ATP-binding protein	*livF*	+	+	+	+	+
Zinc	substrate-binding protein	*znuA*	+	+	+	+	+
Zinc	permease protein	*znuB*	+	+	+	+	+
Zinc	ATP-binding protein	*znuC*	+	+	+	+	+
tungstate	substrate-binding protein	*tupA, vupA*	+	+	+	+	+
tungstate	permease protein	*tupB, vupB*	+	+	+	+	+
tungstate	ATP-binding protein	*tupC, vupC*	+	+	+	+	+
spermidine/putrescine	substrate-binding protein	*potD*	.	.	.	.	+
spermidine/putrescine	permease protein	*potC*	.	.	.	.	+
spermidine/putrescine	permease protein	*potB*	.	.	.	.	+
spermidine/putrescine	ATP-binding protein	*potA*	.	.	.	.	+

Secretion systems

system	Reaction	Gene	*Ca.* H. lyudaonia	*H. aestuarii* DSM 10141	*H. aestuarii* DSM 17919	*H. marinisediminis* DSM 17456	*H. spirochaetisodalis* JC271
Type I secretion system	outer membrane protein	*tolC*	.	.	.	.	.
Type I secretion system	membrane fusion protein	*hlyD, cyaD*	.	+	+	+	+
Type I secretion system	ATP-binding protein	*hlyB, cyaB*	.	+	+	+	+
Type II secretion system	Secretin	*gspD*	+	+	+	+	+
Type II secretion system	outer membrane protein	*gspS*	.	.	.	.	.
Type II secretion system	inner membrane protein	*gspC*	.	+	+	+	+
Type II secretion system	inner membrane protein	*gspF*	+	+	+	+	+
Type II secretion system	inner membrane protein	*gspG*	+	+	+	+	+
Type II secretion system	inner membrane protein	*gspH*	.	.	.	.	.
Type II secretion system	inner membrane protein	*gspI*	+	+	+	+	.
Type II secretion system	inner membrane protein	*gspJ*	+	+	+	+	+
Type II secretion system	inner membrane protein	*gspK*	+	+	+	+	+
Type II secretion system	inner membrane protein	*gspL*	.	.	.	.	.
Type II secretion system	inner membrane protein	*gspM*	+	+	+	.	+
Type II secretion system	ATPase	*gspE*	+	+	+	+	+
Type II secretion system	Leaderpeptidase	*gspO*	.	.	.	.	.

Secretion systems

system	Reaction	Gene	***Ca*. H. lyudaonia**	***H. aestuarii* DSM 10141**	***H. aestuarii* DSM 17919**	***H. marinisediminis* DSM 17456**	***H. spirochaetisodalis* JC271**
Type III secretion system	Needle	*yscF, sctF, ssaG, prgI*	.	.	.	+	+
Type III secretion system	Needle	*yscO, sctO*	+	.	.	.	.
Type III secretion system	Needle	*yscP, sctP*	.	.	.	.	.
Type III secretion system	Needle	*yscX, sctX*	+	.	.	+	.
Type III secretion system	Secretin	*yscC, sctC, ssaC*	+	.	.	+	+
Type III secretion system	outer membrane protein	*yscW, sctW*	+	.	.	+	+
Type III secretion system	inner membrane protein	*yscJ*	+	.	.	+	+
Type III secretion system	inner membrane protein	*yscR*	+	.	.	+	+
Type III secretion system	inner membrane protein	*yscS*	+	.	.	+	+
Type III secretion system	inner membrane protein	*yscT*	+	.	.	+	+
Type III secretion system	inner membrane protein	*yscU*	+	.	.	+	+
Type III secretion system	inner membrane protein	*yscV*	+	.	.	+	+
Type III secretion system	ATPase	*yscN*	.	.	.	.	.
Type III secretion system	ATPase-associated protein	*yscQ*	.	.	.	.	.
Type III secretion system	ATPase-associated protein	*yscL*	+	.	.	+	+
Type VI secretion system	secreted substrates	*vgrG*	+	+	.	.	.
Type VI secretion system	secreted substrates	*hcp*	+	+	.	.	.
Type VI secretion system	outer membrane protein	*vasD*	+	+	.	.	.
Type VI secretion system	inner membrane protein	*impL*	+	+	.	.	.
Type VI secretion system	inner membrane protein	*impK*	+	+	.	.	.
Type VI secretion system	ATPase	*vasG*	+	+	.	.	.
Type VI secretion system	regulatory proteins	*ppkA*	.	.	.	.	.
Type VI secretion system	regulatory proteins	*fha1*	.	.	.	.	.
Type VI secretion system	regulatory proteins	*stp1*	.	.	.	.	.

system	Reaction	Gene	***Ca*. H. lyudaonia**	***H. aestuarii* DSM 10141**	***H. aestuarii* DSM 17919**	***H. marinisediminis* DSM 17456**	***H. spirochaetisodalis* JC271**
Twin-arginine translocation pathway	inner membrane protein	*tatA*	+	+	+	+	+
Twin-arginine translocation pathway	inner membrane protein	*tatB*	+	+	+	+	+
Twin-arginine translocation pathway	inner membrane protein	*tatC*	+	+	+	+	+
Twin-arginine translocation pathway	inner membrane protein	*tatE*	.	.	.	.	.
General secretory pathway	inner membrane protein	*secD*	+	+	+	+	+
General secretory pathway	inner membrane protein	*secE*	+	+	+	+	+
General secretory pathway	inner membrane protein	*secG*	+	+	+	+	+
General secretory pathway	inner membrane protein	*secY*	+	+	+	+	+
General secretory pathway	inner membrane protein	*yajC*	+	+	+	+	+
General secretory pathway	inner membrane protein	*yidC, spoIIIJ, OXA1, ccfA*	+	+	+	+	+
General secretory pathway	ATPase	*secA*	+	+	+	+	+
General secretory pathway	Secretion Monitor	*secM*	.	.	.	.	.
General secretory pathway	SRP receptor	*ftsY*	+	+	+	+	+
General secretory pathway	Targeting protein	*secB*	.	.	.	.	+
General secretory pathway	Targeting protein	*SRP54, ffh*	+	+	+	+	+
